# Editorial: Advances in Musculoskeletal Modeling and Their Application to Neurorehabilitation

**DOI:** 10.3389/fnbot.2020.00065

**Published:** 2020-10-09

**Authors:** Mohammad S. Shourijeh, Naser Mehrabi, John J. McPhee, Benjamin J. Fregly

**Affiliations:** ^1^Rice Computational Neuromechanics Laboratory, Department of Mechanical Engineering, Rice University, Houston, TX, United States; ^2^General Motors Company, Canadian Technical Centre, Markham, ON, Canada; ^3^Motion Research Group, Systems Design Engineering, University of Waterloo, Waterloo, ON, Canada

**Keywords:** musculoskeletal modeling, rehabilitation, neuromechanics, neurorehabilitation, neuromusculoskeletal modeling, assistive devices, predictive dynamic simulation, biomechatronics

## Introduction

Neuromusculoskeletal (NMS) modeling has the potential to serve as a valuable tool for informing the design of assistive devices or neurorehabilitation interventions. For these applications, an NMS model must be able to represent the relevant unique characteristics of the targeted subject, e.g., a person with a neurological disorder. Motion predictive simulations, by considering the task constraints and the neuromuscular requirements, can predict novel movements. Such simulation methods must ideally capture the features and functionalities of the motor control system to mimic human neuromechanical behavior. If a simulation performed with an NMS model fails to accurately predict how a person moves or how that person controls his or her muscles during movement, it will have limited utility for informing device and intervention design. Although, in the past few years, several studies have developed NMS models and techniques to anticipate responses to interventions, e.g., orthopedic surgery, orthoses, or neurorehabilitation, a well-established rigorous framework that can accurately predict neuromusculoskeletal dynamics of healthy and pathologic individuals is still lacking in the research community. Even after the published papers of this topic, there is a strong need for greater research effort in the area of NMS model personalization and personalized simulations of treatment design.

One of the emerging application areas for personalized NMS models is in the design of assistive devices for rehabilitation and augmentation purposes. Integrated human and device interaction models would save time and cost of multiple prototyping steps in both the design and evaluation phases of such devices. Bio-fidelic predictive NMS models could delineate crucial parameters of a design and elucidate the effect of a device conceptual design on an individual's kinematics and kinetics. Therefore, the design process will become human-centered in which a design optimization step includes all important factors, such as mechanical, electrical, material, as well as physiological.

The perspective of this Research Topic was to cover the following topics by taking advantage of neuromusculoskeletal models:

1. New methods and approaches in the field of musculoskeletal modeling and simulation, including motion prediction, neural control, central pattern generators, subject-robot co-simulations, foot-ground contact dynamics, subject-specific simulations
2. Simulated effects of neurological conditions such as stroke, spinal cord injury, and cerebral palsy on neural control and muscle properties
3. Design of human-centered assistive devices such as prostheses, orthoses, and hard and soft exoskeletons by investigating properties such as kinematic alignment, changes in subject movement pattern, strap and joint reaction forces, and metabolic energy consumption
4. Studies of human-robot interaction.

The 10 contributions to this Research Topic provide a wide range of perspectives and methodologies. These high-quality contributions highlight the present potential of a community of researchers interested in an interdisciplinary view of the interaction between neuromusculoskeletal modeling and rehabilitation science to compensate, augment, and restore human function.

## The Contributions

Many contributions to this topic combine experimental data with theoretical approaches that were directly related to neurorehabilitation (Pitto et al.; Romero-Sánchez et al.; Sauder et al.) or can provide a better understanding of human neuromechanics for future treatment design (Millard and Mombaur; Seth et al.; Shuman et al.; Moissenet et al.). Experimental human data spanned kinematics from surface markers, ground reaction forces from force plates or instrumented treadmills, and extensive fine wire and surface EMG for muscles. Theoretical techniques ranged from kinematic synergy analyses (Tang et al.) to highly complex predictive neuromusculoskeletal optimal control problems (Sauder et al.; Pitto et al.). Most of the contributions included a form of musculoskeletal modeling/simulation, either by developing a new method (Sauder et al.) or adapting a previously developed technique (.Moissenet et al.; Tang et al.). The assistive devices in the contributed studies ranged from passive orthoses (Michaud et al.) to hybrid active exoskeletons (Romero-Sánchez et al.). There was also a narrative review paper that presented interesting ideas and concepts for the next generation of neuroprostheses (Pizzolato et al.). Overall, the contributions to this Research Topic reveal the wide range of research being conducted in neuromusculoskeletal (NMS) modeling and the essential role it can play in the future of neurorehabilitation.

Below, an overview is provided of the scientific contributions to this Research Topic, which are listed within two main categories:

1) Development of new NMS modeling methods that contribute to the future of rehabilitation treatment design

2) Application of existing NMS modeling methods to rehabilitation treatment design.

## Development of New NMS Modeling Methods that Contribute to the Future of Rehabilitation Treatment Design

There are two important challenges in using NMS models in the design process of neurorehabilitation. The first challenge is to personalize generic NMS models to the relevant unique characteristics of the patient, including personalization of the neuromechanical impairment. The second challenge is to simulate the effect of different treatment scenarios on movement performance. Two contributions to this topic (Sauder et al.; Pitto et al.) address these two challenges. Both studies used OpenSim as their musculoskeletal modeling package, modeled neural coordination through the concept of muscle synergies, used pre-treatment data for model personalization, and assumed that some components of the neural coordination stay the same after treatment. Sauder et al. improved a model personalization and treatment optimization pipeline for enhancing the efficiency of fast functional electrical stimulation. Optimal control simulations showed that optimizing muscle selection and stimulation timing results in a 23% improvement in the gait symmetry of an individual post-stroke compared to the simulated results from common clinical practice. Pitto et al. developed a simulation pipeline to explore the effect of treatment by changing muscle and geometry parameters in cerebral palsy children. A graphical user interface was also developed for modifying the musculoskeletal properties according to the surgical plan. Both studies are new doors to the use of predictive NMS modeling for the design and evaluation of treatment.

Foot-ground contact model is an essential element in predictive NMS models of gait. Although the foot-ground interaction is often modeled as a grid of viscoelastic elements, Millard and Mombaur developed and evaluated two rigid foot-ground contact models that have potential advantages, e.g., model calibration is reduced to a geometric problem, and the numerical stiffness of the equations of motion is similar in both swing and stance phase. Both the ellipse-foot and the double-circle-foot models were evaluated by analyzing ankle angle and the center-of-pressure (CoP) kinematics, accuracy of kinematics in a tracking gait problem, and changes in kinematics during a predicted gait problem. Each model showed pros and cons with the overall capability for use in predictive gait simulations.

To examine muscle function and to predict movement reliably, one must use physiologically plausible musculoskeletal models. The upper extremity is usually affected in neurological impairments such as stroke, and the shoulder is one of the key joints possessing a complex structure as well as a load-carrying role. Seth et al. developed a novel shoulder model and looked at the contribution of work and movement of muscles to shoulder movement. Previous models of the human shoulder had coupled scapula movement to humeral movement. To elucidate the roles of the thoracoscapular muscles, Seth et al. developed a shoulder model that represents the scapulothoracic joint accurately and includes scapular muscles. The authors also showed that the large thoracoscapular muscles do more work than glenohumeral muscles during arm-elevation tasks. Therefore, this model, which along with the experimental data is freely available on SimTK.org, is expected to be applied by researchers, including NMS modelers designing treatments for the shoulder joint.

One of the challenges in the design of active assistive devices is the harmony between the device actuators and the individual's desired natural movement. Romero-Sánchez et al. developed a theoretical framework and a computational model for a hybrid orthosis—also referred to as a rehabilitation exoskeleton, which consists of electromechanical actuation and functional electrical stimulation (FES). The main challenge in these devices is to coordinate and make transparent the two actuators governing each degree of freedom so that their actuation profiles lead to a natural motion, e.g., gait. Romero-Sánchez et al. included muscular dynamics to attain higher neurophysiological fidelity for the rehabilitation purpose for which these devices are designed. This integrated approach estimates the actuation profiles so that the reduction in peak muscle force due to FES-inducing fatigue is compensated for a hybrid hip-knee-ankle-orthosis as the assistive device. It is hoped that the study's results and theoretical workflow will contribute to the design of more physiological hybrid exoskeletons that can maintain rehabilitative function of the device by improving its actuation control.

## Application of Existing NMS Modeling Methods to Rehabilitation Treatment Design

Moissenet et al. established a computational approach suitable for tracking simulations of healthy and pathological gait. The approach can track experimental data while also allowing for simulation of different variations in the model, e.g., pathology or treatment. A previously developed EMG-marker tracking optimization method was adapted to a lower extremity musculoskeletal model during equinus gait. This dynamic optimization approach tracked experimental marker trajectory, EMG signal, and ground reaction force data. Although this preliminary study was the authors' first step toward their treatment modeling framework, this approach showed its potential to be a candidate for experimentally and dynamically consistent musculoskeletal simulations.

One key aspect of neuromusculoskeletal modeling is muscle recruitment or force distribution problem. It has been hypothesized that the human central nervous system simplifies the construction of muscle excitations during dynamic tasks by constraining those excitations to weighted groups, referred to as muscle synergies. In this Research Topic, Pitto et al., Sauder et al., and Shuman et al. applied muscle synergies in different NMS simulations to address populations with different neurological disorders. While Sauder et al. targeted stroke, Pitto et al. and Shuman et al. looked at or cerebral palsy (CP). While Sauder et al. and Pitto et al. predicted gait post-treatment, Shuman et al. focused on estimating muscle activations and discussing whether estimates generated by imposing a muscle synergy structure improve the correlation with experimental muscle activities. Shuman et al. used a scaled generic musculoskeletal model and compared two cases: (1) static optimization (SO) with minimization of squared muscle activations, and (2) synergy SO (SynSO) with minimization of squared synergy activations and using synergy weights obtained by analysis of EMG data. In SynSO, synergy weights are decomposed from EMG data while the synergy activations are computed one time step at a time, similar to SO. The correlation with EMG data was not found to be higher in SynSO than in SO.

It is a kinematic requirement that rehabilitation exoskeletons move with a spatiotemporal motion that is synchronized with the kinematic structure of the upper-limb joint. Tang et al. analyzed the spatiotemporal kinematic synergies of arm reaching movements and investigated their potential usage in upper limb assistive exoskeleton motion planning. Kinematic synergies—coordination between shoulder and elbow joints—were extracted by running principal component analysis on experimental reaching trials of multiple subjects. Tang et al. concluded that kinematic synergies could be used for exoskeleton motion planning, and different principal components partly contributed to motion trajectory and end-point accuracy. Although this study did not include a kinetic and a neuromuscular model, the concept of kinematic synergies may provide simplified yet worthwhile strategies for initial motion planning and kinematic design of assistive devices to restore natural upper-limb motion and motor function.

Energy expenditure is one of the key criteria used for the design and evaluation of assistive devices in different populations. While total body metabolic energy rate can be measured by means of O_2_/CO_2_ consumption/production rates experimentally, muscle metabolic models could be utilized for two main reasons: (1) It is nearly impossible today to measure the metabolic energy of a muscle experimentally and (2) There may be circumstances in which it is desirable to avoid *in vivo* measurement of subjects or activities. Michaud et al. adapted their previously proposed musculoskeletal modeling approach for estimating muscle forces/activations to a healthy subject and validated their total body metabolic energy rate estimates from two commonly used models with data from a portable gas analyzer. Then, both metabolic energy rate models estimated energetic efficiency using two types of assistive devices on an individual with spinal cord injury during crutch-assisted gait. The two assistive devices were a passive and an active knee-ankle foot orthosis (KAFO). The authors found that the active KAFO resulted in simultaneous improved gain pattern and reduced energy consumption.

Pizzolato et al. presented a narrative review of how NMS models combined with finite element models of musculoskeletal tissues can be integrated with models of assistive and robotic devices for neural restoration in the SCI population. The authors discussed how NMS models can be deployed in related real-time applications, such as muscle activity optimization, functional progress tracking, balance and safety monitoring, and augmented feedback during rehabilitation. Enhancement of current neurorehabilitation may be achieved via development of the next generation orthoses/prostheses incorporating personalized NMS models of patients.

## Conclusions

This Research Topic focuses on advances in neuromusculoskeletal modeling and applications to neurorehabilitation. Contributions to this topic utilized both experimental and computational approaches to address research questions involving neuromechanics and rehabilitation. The researchers contributed to the topic and the editors hope that this Research Topic will open new horizons to more direct clinical applications of neuromusculoskeletal modeling. Although research studies of high quality were presented under this Research Topic, we hope that these studies will only be the beginning and that momentum would continue to grow in this research area. We eagerly hope to observe more advances in neuromusculoskeletal model personalization and their application toward personalized interventions to restore, compensate, or augment motor function, such as via gait retraining or design of assistive devices, in neurologically impaired populations.

## Author Contributions

All editors of this Research Topic, i.e., MSS, NM, JJM, and BJF, have made a significant contribution to this work and approved it for publication.

## Conflict of Interest

NM was employed by the company General Motors. The remaining authors declare that the research was conductedin the absence of any commercial or financial relationships that could be construed as a potential conflict of interest.

